# Computational models of basal-ganglia pathway functions: focus on functional neuroanatomy

**DOI:** 10.3389/fnsys.2013.00122

**Published:** 2013-12-30

**Authors:** Henning Schroll, Fred H. Hamker

**Affiliations:** ^1^Bernstein Center for Computational Neuroscience, Charitè – Universitätsmedizin BerlinBerlin, Germany; ^2^Department of Psychology, Humboldt-Universität zu BerlinBerlin, Germany; ^3^Department of Neurology, Charitè – Universitätsmedizin BerlinBerlin, Germany; ^4^Department of Computer Science, Chemnitz University of TechnologyChemnitz, Germany

**Keywords:** dopamine, reinforcement learning, response selection, response timing, working memory, gating, stimulus-response association

## Abstract

Over the past 15 years, computational models have had a considerable impact on basal-ganglia research. Most of these models implement multiple distinct basal-ganglia pathways and assume them to fulfill different functions. As there is now a multitude of different models, it has become complex to keep track of their various, sometimes just marginally different assumptions on pathway functions. Moreover, it has become a challenge to oversee to what extent individual assumptions are corroborated or challenged by empirical data. Focusing on computational, but also considering non-computational models, we review influential concepts of pathway functions and show to what extent they are compatible with or contradict each other. Moreover, we outline how empirical evidence favors or challenges specific model assumptions and propose experiments that allow testing assumptions against each other.

## 1. Introduction

### 1.1. Introduction to the concept of basal-ganglia pathways

Basal ganglia (BG) contain a variety of both glutamatergic and GABAergic fiber tracts. Why is BG organization that complex? Two influential theories, published more than 20 years back (Albin et al., 1989; DeLong, 1990), came up with a first idea: they proposed that BG control excitation and inhibition of cortex, therefore requiring two distinct pathways: a direct pathway (cortex→striatum→globus pallidus internus) was assumed to facilitate motor cortical activity, while an indirect pathway (cortex→striatum→globus pallidus externus→subthalamic nucleus→globus pallidus internus) was assumed to inhibit motor-cortical firing. These concepts provided an explanation of prominent BG motor disorders: over-activity of the excitatory direct pathway was assumed to result in overshoot of motor activity (as in Huntington's disease), while over-activity of the inhibitory indirect pathway was proposed to result in pathological motor inhibition (as in Parkinson's disease; Albin et al., 1989; DeLong, 1990). Inspired by this intuitive concept and the fact that it was later discovered to fail at explaining some prominent empirical findings (e.g., Marsden and Obeso, 1994), revised and extended models have been developed since. As part of this process, an additional, shorter route of the indirect pathway (cortex→striatum→globus pallidus externus→globus pallidus internus) has been proposed (Smith et al., 1998) as well as an additional hyperdirect pathway (cortex→subthalamic nucleus→globus pallidus internus; Nambu et al., 2002). If all of these pathways can be identified to fulfill distinct functions, the complexity of BG anatomy might be understood as a necessity to guarantee BG functionality.

**Figure 1:**
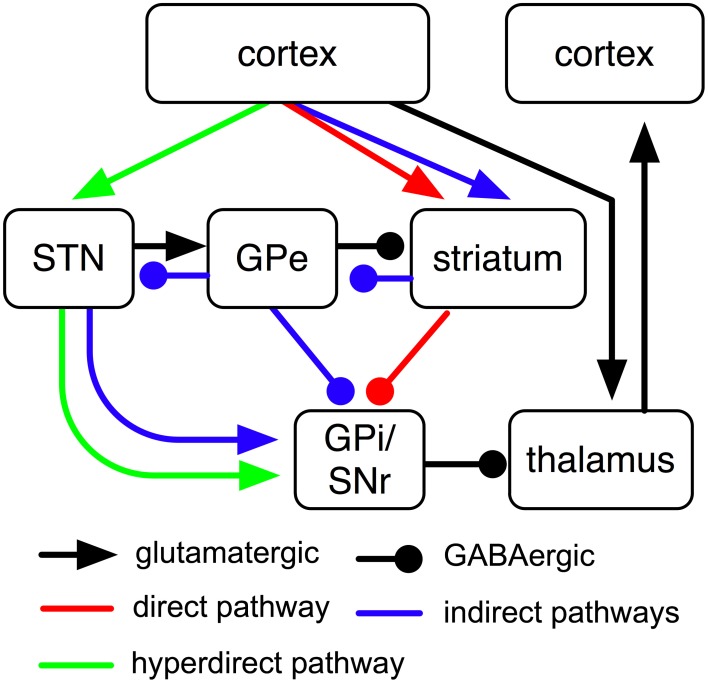
**Sketch of cortico-BG-thalamic fiber tracts and their subdivision into direct, indirect and hyperdirect BG pathways (cf. Bolam et al., 2000).** Of the “indirect pathway,” two routes have been proposed (Smith et al., 1998), the short one of which passes from GPe directly to GPi, while the longer one additionally passes through STN.

According to general understanding, direct, indirect and hyperdirect BG pathways transmit cortical input to globus pallidus internus (GPi) and substantia nigra reticulata (SNr), two largely analog BG output nuclei that tonically inhibit the thalamus (Figure 1). The direct pathway proceeds from cortex via striatum to GPi; information traversing this pathway has to pass a glutamatergic synapse first and a GABAergic synapse afterwards (Figure 1). Cortical input to the direct pathway thus reduces GPi firing which in turn increases activities in thalamus and cortex. The short indirect pathway passes from cortex to GPi via striatum and globus pallidus externus (GPe); synapses are glutamatergic, GABAergic and GABAergic, respectively. The *long* indirect pathway, in contrast, additionally passes through the subthalamic nucleus (STN) and contains an additional glutamatergic synapse (Figure 1). Cortical input to either of the two indirect pathways thus increases GPi firing. The hyperdirect pathway, finally, passes from cortex via STN to GPi and contains glutamatergic synapses only; cortical input to this pathway therefore increases GPi activity as well. Pathways are usually assumed to transmit information in a feed-forward manner; existing feedback-projections (e.g., from GPe to striatum or from STN to GPe; cf. Figure 1) are either assumed to not be part of these pathways or are assumed to be required for stabilization of information transmission only.

In the last decade, the rise of computational simulation techniques has boosted model development. Today, there is a multitude of different models, none of which yet accounts for all relevant empirical findings (section 9). Most of these models assume a clear anatomical separation between the different pathways. Although this is likely a simplification (Lévesque and Parent, 2005), physiological data corroborates the assumption of functionally separate pathways: electrical stimulation of cortex results in three temporally distinct changes of activity in GPi that can be traced back to the effects of direct, indirect and hyperdirect pathways, respectively (Nambu et al., 2000; Kita et al., 2006; Kita and Kita, 2011). Even if pathways are not built out of distinct sets of neurons, thus, they appear to be functionally separated.

### 1.2. Why computational modeling?

Most of the models and hypotheses we will review offer not just verbal and graphical descriptions, but an additional mathematical (i.e., computational) implementation. Such mathematical implementations offer important advantages, including, but not limited to the following: they allow computing the effects of non-linear interactions between simulated neurons that would be impossible to compute mentally. Moreover, they are innately precise, thus preventing fuzzy assumptions; if some of a model's various assumptions contradict each other or do not interact well, the model will fail to produce meaningful output. Finally, computational models produce predictions that do not immediately originate from their assumptions. Such predictions might, for instance, relate to model performance during specific behavioral tasks. As a note of caution, however, computational models are often hard to grasp intuitively: a set of mathematical formulas does not innately reveal what function a model serves. Rather, extensive and often iterative simulations are required to reveal these functions. To report and review computational models, thus, verbal and graphical descriptions of model assumptions and outputs are required as well. These, however, may suffer from lack of precision and in any case simplify a model's “real” computational details.

In the context of BG functioning, computational modeling has been particularly fruitful in recent years. The complexity of BG anatomy and physiology, in light of their substantial interactions with cortex, thalamus and other sub-cortical nuclei makes them a good target for computational modeling.

## 2. Anatomical and physiological constraints for interpretations of pathway functions

### 2.1. Pathway afferents from cortex and thalamus

**Figure 2:**
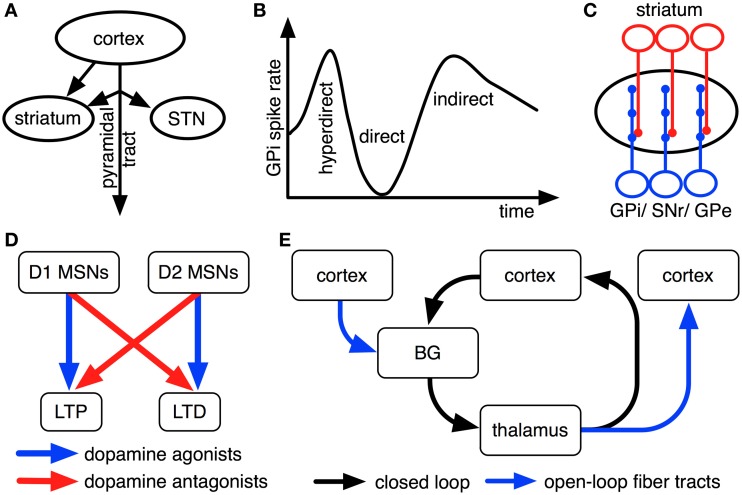
**Empirical findings that constrain interpretations on pathway functions.** For illustrative purposes, all findings are shown as cartoons. **(A)** Striatum receives inputs from both intratelencephalically projecting cortical neurons and cortical pyramidal-tract neurons, while STN predominantly receives pyramidal-tract afferents (Donoghue and Kitai, 1981; Giuffrida et al., 1985; Lei et al., 2004; Parent and Parent, 2006; Kita and Kita, 2012). **(B)** Upon electrical stimulation of cortex, direct, indirect and hyperdirect pathways influence GPi activity with different latencies because of their different conduction velocities (Nambu et al., 2000; Kita and Kita, 2012). **(C)** Striatal cells innervate relatively small, circumscribed areas of GPi, SNr and GPe dendrites, whereas STN cells innervate these nuclei relatively broadly (Hazrati and Parent, 1992a,b). **(D)** Dopamine agonists and antagonists oppositely modulate long-term plasticity in cortico-striatal synapses of direct and indirect pathways (cf. Shen et al., 2008). **(E)** BG are organized in open and closed loops with cortex and thalamus (cf. Alexander et al., 1986; Joel and Weiner, 1994; Haber, 2003).

The striatum (which is part of direct and indirect pathways) receives topographically organized inputs both from intratelencephalically-projecting cortical cells and from axon collaterals of cortical pyramidal-tract neurons (Figure 2A; Donoghue and Kitai, 1981; Lei et al., 2004; Parent and Parent, 2006; Shepherd, 2013). Cortico-striatal cells are predominantly located in cortical layer V, but also in layers II, III, and IV (Rosell and Giménez-Amaya, 1999). Striatal medium spiny neurons (MSNs) of the direct pathway have been shown to receive the majority of their inputs from intratelencephalically projecting cortico-striatal neurons, while striatal MSNs of the indirect pathways receive a greater proportion of inputs from axon collaterals of cortical pyramidal-tract neurons (Lei et al., 2004). The indirect pathways' inputs might thus largely consist of efference copies of motor output, informing this pathway about currently initiated responses.

Next to its MSNs, striatum contains cholinergic and several types of GABAergic interneurons (Tepper, 2010). While GABAergic interneurons receive extensive cortical input (Lapper et al., 1992; Kawaguchi, 1993; Ramanathan et al., 2002), cholinergic interneurons might receive more extensive input from thalamus than from cortex (Lapper and Bolam, 1992; Kawaguchi, 1993). Thalamic efferents to striatum are extensive and topographically organized (Berendse and Groenewegen, 1990; Lanciego et al., 2004).

STN (which gives rise to the hyperdirect pathway) receives topographically organized inputs from frontal and motor cortices (Hartmann-von Monakow et al., 1978; Afsharpour, 1985), again mainly from layer V (Canteras et al., 1990). Its cortical afferents have been described as deriving mainly from axon collaterals of cortico-fugal pyramidal-tract neurons (Giuffrida et al., 1985; Kita and Kita, 2012), thus potentially providing STN with efference copies of motor output. Potential inputs from sensory cortical areas have been both reported (Canteras et al., 1988) and repudiated (Afsharpour, 1985; Kolomiets et al., 2001). In any case, sensory cortices may modulate STN activity multi-synaptically via striatum and GPe of the long indirect pathway (Kolomiets et al., 2001). Like the striatum, STN receives topographically organized inputs from thalamus (Lanciego et al., 2004).

### 2.2. Pathway conduction velocities

Electrical stimulation of motor cortex results in triphasic changes of activity in GPi (Figure 2B; Nambu et al., 2000). Approximately 8 ms after stimulation of primary motor cortex, a fast excitation of GPi is observed that is followed by a short inhibition at about 21 ms after stimulation and a late excitation at about 30 ms after stimulation on average (Nambu et al., 2000). Chemical blocking of BG nuclei as well as parallel recordings in STN and GPe have shown that the fast excitation is caused by the hyperdirect pathway, the short inhibition by the direct pathway and the late excitation by the long indirect pathway (Nambu et al., 2000; Kita et al., 2006; Kita and Kita, 2011). The hyperdirect pathway's exceptionally fast response has been linked to unique properties of STN neurons, involving a slow decay of excitatory postsynaptic potentials (EPSPs) and a dynamic decrease in spike threshold after EPSPs (Farries et al., 2010; Kita and Kita, 2011).

### 2.3. Arborization patterns of pathway outputs

BG pathways arborize differently broadly in GPi, thus affecting different numbers of GPi neurons: striatal neurons arborize with a high degree of specificity in globus pallidus in monkeys (Hazrati and Parent, 1992b), while STN neurons more uniformly excite large numbers of pallidal cells (Hazrati and Parent, 1992a,b; Figure 2C). Despite their different patterns of arborization, however, striatal and subthalamic cells were found to converge onto the same pallidal neurons in internal and external segments of globus pallidus (Hazrati and Parent, 1992a,c). Based on this evidence, the direct pathway and the short indirect pathway are usually assumed to influence relatively focused pallidal representations, whereas the hyperdirect pathway and the long indirect pathway likely exert relatively global effects (cf. Mink, 1996; Nambu et al., 2002; Brown et al., 2004; Nambu, 2004; Frank, 2006).

### 2.4. Dopaminergic impacts on BG pathways

Synaptic plasticity in BG pathways is modulated by dopamine (Shen et al., 2008): while dopamine facilitates long-term potentiation (LTP) in cortico-striatal synapses of the direct pathway via D1-type dopamine receptors, it facilitates long-term depression (LTD) in cortico-striatal synapses of the indirect pathways via D2-type dopamine receptors (Figure 2D; Shen et al., 2008). Phasic BG dopamine signals, as emitted by neurons of substantia nigra compacta (SNc), have been hypothesized to encode error signals of reward prediction (Hollerman and Schultz, 1998): whenever an animal receives more reward than could be expected based upon previous reinforcement contingencies, dopamine neurons increase their firing above a low baseline rate; whenever less reward is received than could have been expected, firing decreases below this baseline. These findings inspired proposals that BG play an important role in reinforcement learning processes in the brain. Dopamine neurons, moreover, do not exclusively respond to rewarding events, but presumably also to salient non-rewarding and to aversive events (Bromberg-Martin et al., 2010). Dopaminergic effects on synaptic plasticity are well studied only for cortico-striatal synapses of direct and indirect pathways (Gerfen et al., 1990; Shen et al., 2008). For striatal outputs to GPe, GPi, and SNr, in contrast, the effects of dopamine have not yet been studied in similar detail. There are, however, hints that dopamine might modulate synaptic plasticity in these nuclei as well: all of them are innervated by axons of SNc dopamine neurons (Cossette et al., 1999; Gauthier et al., 1999); oral administration of the dopamine precursor levodopa modulates activity-dependent synaptic plasticity in SNr (Prescott et al., 2009). Moreover, it has been shown that SNr and entopeduncular nucleus (rat GPi equivalent) predominantly express D1 dopamine receptors (Boyson et al., 1986; Levey et al., 1993), that globus pallidus (rat GPe equivalent) expresses relatively high quantities of D2 receptors, but probably still more D1 dopamine receptors (Boyson et al., 1986; Levey et al., 1993) and that STN expresses both D1-type and D2-type receptors in considerable quantities (Flores et al., 1999). For connections from STN to SNr, moreover, D1 receptor agonists have been found to increase excitatory postsynaptic currents (EPSCs), whereas D2 receptor agonists decrease them (Ibañez-Sandoval et al., 2006). Based on these pieces of evidence, it has been assumed that increases in dopamine levels facilitate LTP along the entire direct pathway (involving both cortico-striatal and striato-GPi/ striato-SNr synapses), LTD along the entire short indirect pathway and LTP along the entire hyperdirect pathway (Schroll et al., 2013). Although this interpretation is consistent with existing empirical data, it has not yet been proven directly.

It is generally assumed that dopamine exerts additional short-term effects on striatal activity that are in line with dopamine's effects on long-term plasticity: high dopamine levels are assumed to excite D1 MSNs of the direct pathway, while low dopamine levels are hypothesized to excite D2 MSNs of the indirect pathways (e.g., Wichmann and DeLong, 1996; Frank et al., 2004; Frank, 2005). Additionally, dopamine is assumed to modulate MSNs' sensitivity to glutamatergic synaptic inputs from cortex, again oppositely for D1 and D2 MSNs (Humphries et al., 2009). Empirically, dopamine has indeed been shown to modulate ion-channel conductances in the striatum (Calabresi et al., 1987; Lin et al., 1996). If, however, dopamine in fact spontaneously excites the direct and inhibits indirect pathways, remains to be shown (Calabresi et al., 2007). Similarly, it needs to be clarified to what extent spontaneous effects of dopamine fulfill a behaviorally relevant function on their own or might simply support dopaminergic effects on long-term plasticity. A detailed model of how dopamine affects the membrane properties of striatal MSNs has been provided by Humphries et al. (2009). Extending their model by known effects of dopamine on synaptic plasticity and including it, as a module, in systems-level models of cortico-BG-thalamic circuitry might help to understand the complex effects of dopamine on the functions of BG pathways.

### 2.5. Cortico-BG-thalamic loops

As outlined in Figure 1, BG are organized in loops with cortex and thalamus. It has been proposed that separate cortico-BG-thalamic loops work in parallel and in relative independence of each other (Alexander et al., 1986). The number of independent loops is hard to estimate. Alexander et al. (1986) proposed the existence of at least five such loops (corresponding to motor, oculomotor, dorsolateral prefrontal, lateral orbitofrontal and anterior cingulate cortex). Frank et al. (2001) later suggested that each of these loops might be again subdivided into various sub-loops and estimated the human frontal cortex to contain around 20,000 such loops in total. Interestingly, the assumption of independent loops implies that each BG pathway has a variety of separate channels (i.e., one for each loop) and that each of these channels might subserve a different function (cf. Schroll et al., 2012). Thus, it might be more fruitful to search for superordinate principles of pathway functions than for specific pathway contributions related to individual loops. Along these lines, it has been distinguished between open and closed cortico-BG-thalamic loops (Figure 2E; Alexander et al., 1986; Joel and Weiner, 1994; Haber, 2003): while closed loops connect a particular area of cortex back to that same cortical area, open loops interconnect different areas of cortex. Anatomical crossovers between loops have indeed been described, in particular for cortico-striatal synapses (e.g., Inase et al., 1996; Takada et al., 1998; Calzavara et al., 2007) and cortico-thalamic synapses (Darian-Smith et al., 1999; McFarland and Haber, 2002). BG pathways might have entirely different functions in open loops than in closed loops. In particular, closed loops appear well fit for maintenance of information, while open loops might foster spread of information between cortical areas (Schroll et al., 2012; Trapp et al., 2012). For open loops, a hierarchy of information flow has been proposed that favors transmission of information from motivational via cognitive toward motor loops, but not vice versa (Haber, 2003).

## 3. Hypotheses on functional contributions of basal ganglia

The above-mentioned pieces of evidence restrict the degrees of freedom for plausible hypotheses on pathway functions, but still leave a lot of interpretive freedom. Before reviewing hypothesized contributions of individual pathways, we will outline proposed functions of BG as an entirety.

### 3.1. Selection machine

**Figure 3:**
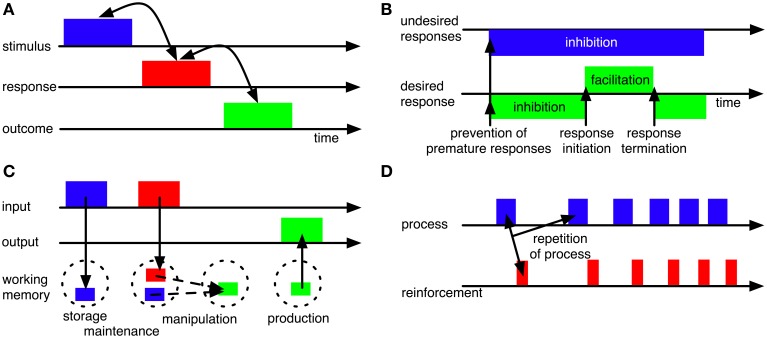
**Some influential concepts of BG functions. (A)** BG may establish and maintain associations between stimuli and responses (or even between stimuli, responses and outcomes; Redgrave and Gurney, 2006) to allow context-based response selection. **(B)** BG may contribute to motor timing by providing initiation and termination signals for movements (Nambu, 2004) and by inhibiting premature responding (Frank, 2006). **(C)** BG may contribute to working memory functions, including gating of information into working memory (Gruber et al., 2006; O'Reilly and Frank, 2006), working-memory maintenance (Schroll et al., 2012) and production of information from working memory (Schroll et al., 2012). **(D)** BG may contribute to reinforcement learning processes, including, but not limited to reward-based learning, such that those processes or actions that result in reinforcements will be repeated (e.g., Houk et al., 1995; Berns and Sejnowski, 1998; Suri et al., 2001).

BG have been hypothesized to contribute to the selection of motor responses (e.g., Mink, 1996; Hikosaka et al., 2000; Gurney et al., 2001a,b; Frank et al., 2004; Nambu, 2004; Ashby et al., 2007; Schroll et al., 2012). Allowing for context-appropriate selection, they have moreover been assumed to establish and maintain associations between stimulus representations and response representations (Figure 3A; e.g., Reading et al., 1991; Packard and Knowlton, 2002). In line with these hypotheses, patients with BG disorders (i.e., Parkinson's disease and Huntington disease) are impaired in response selection (Lawrence et al., 1999; Wylie et al., 2009) and lesions of striatum result in impairments in acquiring stimulus-response rules (e.g., Reading et al., 1991; El Massioui et al., 2007). Recently, BG have been reported to be particularly involved in *learning* of stimulus- response associations, while they might be less important for execution of habitual stimulus-response behavior (Antzoulatos and Miller, 2011; Waldschmidt and Ashby, 2011).

In a generalization of the selection hypothesis, BG have been proposed to select any cortical representation (rather than just motor programs), including internal cognitive and emotional states, based upon activation of any other representation (Trapp et al., 2012). In another generalization, BG have been assumed to establish associations not only between stimuli and responses, but between stimuli, responses and outcomes (Figure 3A; Redgrave and Gurney, 2006).

### 3.2. Performance of sequences

Based upon the idea that BG encode stimulus-response associations (section 3.1), they have been hypothesized to establish and execute sequences of motor processes by linking each single response of a sequence to its respective predecessor (Berns and Sejnowski, 1998; Nakahara et al., 2001). According to this hypothesis, BG interlink the different elements of a sequence in a stimulus-response manner, such that each performed “response” of a sequence serves as a “stimulus” for the following response. BG thus do not contain a single “overall” representation of each sequence, but an array of individual associations between its subsequent elements.

Evidence on BG involvement in sequence learning and execution has been provided for grooming in mammals (e.g., Berridge and Whishaw, 1992), song production in songbirds (e.g., Brainard and Doupe, 2000; Kao et al., 2005; Ölveczky et al., 2005) and sensorimotor production in humans (Doyon et al., 1997; Boecker et al., 1998).

### 3.3. Response initiation and termination

BG have been hypothesized to provide initiation and termination signals for motor responding (Figure 3B; Nambu, 2004). According to this hypothesis, cortex sends a succession of corollary signals to the different BG pathways that ensure surround-inhibition of (premature) responses, response initiation and response termination, respectively. According to Nambu's (2004) hypothesis, BG determine the timing of already selected responses based on their corollary input signals from cortex (see also Mink, 1996); Nambu (2004), however, did not develop a computational model; we do not know of any such model that implements BG contributions to initiation and termination of motor responses in a loop linking the BG to primary motor cortex (M1).

Nambu's (2004) concept has been inspired by evidence of BG pathways' different conduction velocities as reviewed in section 2.2: stimulation of cortex first results in excitation, then inhibition, and finally again excitation of GPi. These three phases are assumed to correspond to inhibition of (premature) responses, response initiation and response termination, respectively (Nambu, 2004). However, pathway conduction velocities, by themselves, are no convincing proof of Nambu's (2004) assumptions: differences in conduction velocities are minute in magnitude and also relatively inflexible; they do not explain how response timing can be adapted to different contexts. Moreover, pathways not only have different conduction velocities but also receive different inputs that will likely set in at different times (section 2.1). These different set-ins of inputs may be far more decisive for latencies of pathway outputs than the pathways' conduction velocities. In line with this reasoning, Nambu (2004) hypothesized that pathways require temporally distinct corollary inputs from cortex to properly initiate and terminate responses.

### 3.4. Working memory gating and maintenance

In the cognitive domain, BG have been hypothesized to control working-memory processes (Figure 3C). According to one proposal (O'Reilly and Frank, 2006), they guard the gate to working memory and thereby determine which stimuli will be maintained. Learning which stimuli to gate through initially requires random gating of working-memory contents according to this proposal. According to a different proposal (Schroll et al., 2012), BG are part of a working-memory maintenance system, allowing for reverberation of information in cortico-BG-thalamic loops. Here, BG are assumed to both determine which pieces of information enter working memory and to contribute to their actual maintenance. This model does not require an initially random selection of working-memory contents, but relies on shaping to learn complex working-memory paradigms. There is ample evidence on BG involvement in working memory tasks, both from human (Lewis et al., 2004; Alberts et al., 2008; Hershey et al., 2008; Moustafa et al., 2008b; Landau et al., 2009) and animal subjects (Levy et al., 1997). Empirical differentiation between the two hypotheses, however, is not yet possible as will be outlined in section 4.5.

### 3.5. Dimensionality reduction

BG have been proposed to establish a focus on relevant (salient) information by reducing dimensionality of cortical information (Bar-Gad et al., 2000). Based on dopaminergic reinforcement signals, BG are assumed to learn efficient compression of information in such a way that its approximate reconstruction remains possible. In line with empirical data (Nelson et al., 1992; Nini et al., 1995; Bar-Gad et al., 2000), the model by Bar-Gad et al. (2000) predicts that correlations between neuronal activities decrease from cortex to globus pallidus. It might moreover explain why cortex contains far more neurons than striatum, which again contains far more neurons than GPi/SNr (Oorschot, 1996).

### 3.6. Reinforcement learning

Most computational models assume that BG pathways contribute to reinforcement learning (e.g., Berns and Sejnowski, 1998; Brown et al., 2004; Frank, 2006; O'Reilly and Frank, 2006; Ashby et al., 2007; Stocco et al., 2010; Schroll et al., 2012). According to this hypothesis, BG adapt behavior in such a way that reinforcements are maximized (Figure 3D). Specifically, they are assumed to foster repetition of those actions, emotions and cognitive processes that result in reinforcements.

Under the umbrella term “reinforcement learning,” BG have been proposed to learn from unexpected rewards (e.g., Suri et al., 2001; Brown et al., 2004; Ashby et al., 2007; Vitay and Hamker, 2010), from punishments (e.g., Frank et al., 2004), and, generally, from unexpected sensory events (Redgrave and Gurney, 2006). The latter generalization implies that BG may learn any novel association between stimuli, actions and outcomes, even if not followed by reward. Via such a mechanism, animals and humans might learn contingencies that are relevant for obtaining positive outcomes in the future: for instance, they might find out that a particular action results in access to a safe sleeping place, even when currently foraging for food (cf. Redgrave and Gurney, 2006).

The reinforcement-learning hypothesis is based on findings that phasic dopamine signals in BG encode error signals of reward prediction (Hollerman and Schultz, 1998) and other salient unexpected events (e.g., Horvitz et al., 1997; Rebec, 1998), where value and salience of events might be signaled by distinct dopamine systems (Bromberg-Martin et al., 2010). The hypothesis is further based on evidence that dopamine modulates synaptic plasticity in BG (Shen et al., 2008). However, synaptic plasticity is well investigated only for cortico-striatal fibers. Some computational models thus limit dopamine-modulated learning processes to these cortico-striatal fibers (e.g., Brown et al., 2004; Ashby et al., 2007; Guthrie et al., 2009; Moustafa and Gluck, 2011), while others, more daringly, assume them to occur along more extensive parts of BG pathways (e.g., Vitay and Hamker, 2010; Schroll et al., 2012). In a particularly strong version of the reinforcement-learning hypothesis, BG refrain from processing as a particular function becomes automatized, i.e., after this function has been reliably learned via reinforcements (Ashby et al., 2007). This hypothesis is corroborated by single cell data from monkeys (Antzoulatos and Miller, 2011) and functional imaging data from humans (Waldschmidt and Ashby, 2011). Automatic functioning has instead been assumed to rely on cortico-cortical or cortico-thalamo-cortical connections (Ashby et al., 2007; Schroll et al., 2013). These connections might allow for faster information transfer because of fewer synaptic contacts than the route through the BG and might thus explain reduced reaction times in automatized tasks (Ashby et al., 2007).

The reinforcement-learning hypothesis is a meta-perspective that is fully compatible with any of the hypotheses outlined in sections 3.1 to 3.5, since it refers to how BG pathways arrive at a particular function and not to what that function is. In fact, all of the hypotheses outlined in sections 3.1 to 3.5 may be correct since BG might flexibly learn to establish exactly those functions that result in reinforcements in a given learning context. Empirical evidence on BG involvement in reinforcement learning is extensive for both animals (e.g., Featherstone and McDonald, 2004; El Massioui et al., 2007; Antzoulatos and Miller, 2011) and humans (e.g., Frank et al., 2004; Tanaka et al., 2004; Schönberg et al., 2007; Moustafa et al., 2008a). Computational models are particularly suitable for formalizing (and then simulating) reinforcement-learning processes because of these processes' iterative nature.

## 4. Proposed functions of the direct pathway

As outlined in sections 2.3 and 2.4, the direct pathway facilitates cortical activity, it is strengthened by dopamine and its arborization is focused rather than divergent. In the following sub-sections 4.1 to 4.6, we will review the direct pathway's proposed functions in detail. Sections 5 and 6 will then cover proposed functions of indirect and hyperdirect pathways. To provide a quick overview, Table 1 summarizes which models interpret which aspects of BG anatomy.

**Table 1:** **Summary of model features and foci**.

	**Authors provide a computational implementation**	**Authors address/implement**			**Authors provide a functional interpretation for**					
		**BG motor functions**	**BG working-memory functions**	**plasticity**	**closed loops**	**open loops**	**the direct pathway**	**the short indirect pathway**	**the long indirect pathway**	**the hyperdirect pathway**
Albin et al., 1989	✗	✓	✗	✗	✗	✗	✓	✗	✓	✗
DeLong, 1990	✗	✓	✗	✗	✗	✗	✓	✗	✓	✗
Mink, 1996	✗	✓	✗	✓	✓	✓	✓	✗	✓	(✓)
Bar-Gad et al., 2000	✓	✗	✗	✓	✗	✓	✓	✗	✗	✗
Suri et al., 2001	✓	✓	✗	✓	✗	✓	✓	✗	✓	✗
Gurney et al., 2001a; Humphries et al., 2006	✓	✓	✗	✗	✗	✓	**n/a**	**n/a**	**n/a**	**n/a**
Nambu, 2004	✗	✓	✗	✗	✓	✗	✓	✗	✓	✓
Brown et al., 2004	✓	✓	✗	✓	✓	✓	✓	✓	✓	✓
Ashby et al., 2005	✓	✗	✓	✓	✓	✗	✓	✗	✗	✗
O'Reilly and Frank, 2006	✓	✗	✓	✓	✗	✓	✓	✓	✗	✗
Frank, 2006; Wiecki and Frank, 2013	✓	✓	✗	✓	✓	✓	✓	✓	✓	✓
Ashby et al., 2007	✓	✓	✗	✓	✗	✓	✓	✗	✗	✗
Stocco et al., 2010	✓	✓	✗	✓	✗	✓	✓	✓	✗	✓
Schroll et al., 2012	✓	✓	✓	✓	✓	✓	✓	✗	✗	✓
Chersi et al., 2013	✓	✓	✗	✓	✓	✓	✓	✗	✗	✓
Schroll et al., 2013	✓	✓	✗	✓	✗	✓	✓	✓	✗	✓

Models differ in which anatomical structures they express hypotheses on. The table gives a condensed overview over the features and foci of different models.

### 4.1. Global motor facilitation

Early non-computational models (Albin et al., 1989; DeLong, 1990) as well as a recent computational model of BG pathways (Stocco et al., 2010) proposed that the direct pathway unspecifically facilitates motor activity. And indeed, it has been confirmed optogenetically that stimulation of striatal MSNs of the direct pathway results in increased locomotion in mice (Kravitz et al., 2010). If this is a (relatively) unspecific effect, however, remains to be shown. The relatively sparse arborization of striatal MSNs in GPi intuitively challenges the global-facilitation hypothesis, although the degree of sparseness cannot yet be interpreted in functional terms and thus is no proof against the hypothesis.

### 4.2. Specific motor facilitation

Mink (1996) proposed that the direct pathway specifically facilitates desired responses (rather than motor activity *per se*). This hypothesis therefore directly contradicts the global-facilitation hypothesis outlined in section 4.1. Recent computational models mostly follow Mink's (1996) suggestion (e.g., Gurney et al., 2001a,b; Suri et al., 2001; Brown et al., 2004; Frank, 2005; Ashby et al., 2007; Schroll et al., 2012) and applied it to cognitive operations as well (e.g., O'Reilly and Frank, 2006; Schroll et al., 2012). Because of the multitude of parallel cortico-BG-thalamic loops (section 2.5), it indeed appears likely that different channels of the direct pathway may simultaneously facilitate different types of representations (Schroll et al., 2012). Most computational models moreover hypothesize that the direct pathway *learns* to facilitate specific cortical representations based on rewards (e.g., Suri et al., 2001; Brown et al., 2004; Frank, 2005; Ashby et al., 2007; Schroll et al., 2012). We do not know of any empirical data that favors the specific-facilitation hypothesis over the global-facilitation hypothesis or vice versa.

### 4.3. Stimulus-response mapping

The direct pathway has been hypothesized to facilitate specific motor programs only if they are appropriate in a given stimulus context (e.g., Brown et al., 2004; Ashby et al., 2007; Vitay and Hamker, 2010; Schroll et al., 2012). This hypothesis is a more specific version of the specific-facilitation hypothesis outlined in section 4.2. It says that the direct pathway connects specific stimulus representations to specific response representations (i.e., motor programs) and then facilitates a particular motor program only if the corresponding stimulus representation is active. The stimulus-response hypothesis relies on the existence of open cortico-BG-thalamic loops that interlink cortical areas involved in stimulus processing with areas involved in motor responding. And indeed, striatal areas that receive inputs from both primary somatosensory and primary motor cortices have been reported (Flaherty and Graybiel, 1993). Clear evidence of open cortico-BG-thalamic loops that connect visual or auditory cortices to primary motor cortex, however, has not yet been shown, although both higher-order visual and higher-order auditory cortices are known to project to striatum (LeDoux et al., 1991; Bordi and LeDoux, 1992; Baizer et al., 1993).

In a generalization of the stimulus-response hypothesis, internal states (like emotions, mental images or abstract cognitive concepts) may serve as stimuli for stimulus-response associations as well. In an even broader generalization, the direct pathway may interlink any two cortical representations (cf. Trapp et al., 2012), potentially in a hierarchy from emotional via motivational, cognitive and premotor to motor regions (Haber, 2003).

Ashby and Crossley (2011) proposed that striatal cholinergic interneurons take part in the establishment of stimulus-response associations. They suggest that these interneurons tonically inhibit striatal MSNs of the direct pathway in the absence of stimulus inputs and that a stimulus-contingent release of inhibition is required for a direct-pathway induced activation of responses.

### 4.4. Temporally precise initiation of responses

Nambu (2004) hypothesized that the direct pathway determines the point in time when a particular response is initiated. According to his concept, the cortex selects an appropriate response and then first sends a corollary signal to the hyperdirect pathway, which globally inhibits all motor programs. Briefly afterwards, a second corollary signal to the direct pathway initiates a specific motor response at the appropriate point in time. This response-initiation hypothesis is, in its core, compatible with the specific-facilitation hypothesis (section 4.2) and the stimulus-response hypothesis (section 4.3): the direct pathway may well select appropriate responses (potentially based upon stimulus input) and also determine the exact time at which they are initiated. The specifics of these hypotheses, however, are incompatible: according to Nambu (2004), the *cortex* decides for a response, while it is the direct pathway and its specific connectivity according to the other two proposals.

Empirical evidence for the response-initiation hypothesis comes from patients with Parkinson's disease, a BG disorder that goes along with decreased activation of the direct pathway (but also with increased activation of the indirect pathways; Kravitz et al., 2010; Kita and Kita, 2011): as predicted by the response-initiation hypothesis, Parkinson's disease patients are impaired in initiating movements (Bloxham et al., 1984; Carli et al., 1985; Hikosaka et al., 1993), but not in completing them (Carli et al., 1985) or in performing pre-programmed movements (Bloxham et al., 1984).

The response-initiation hypothesis as well as, in fact, any of the hypotheses outlined in sections 4.1 to 4.3, has been challenged based on reports that BG activation mostly occurs only *after* overt responses are visible (e.g., Aldridge et al., 1980; Jaeger et al., 1995). However, some BG neurons *do* become active before EMG onset (Jaeger et al., 1995) and reports of delayed BG activity may result from the specific study designs involved: motor responses were trained for extensive amounts of time in these studies, before BG were recorded. Recent evidence, however, points at an important role of the BG in initiating responses only while new response patterns are being *learned* (Antzoulatos and Miller, 2011; Waldschmidt and Ashby, 2011).

### 4.5. Working-memory gating and maintenance

The direct pathway has been suggested to play an important role in working-memory functions. In particular, it has been suggested to gate information into working memory (Gruber et al., 2006; O'Reilly and Frank, 2006), but also to contribute to working-memory maintenance itself (Ashby et al., 2005; Schroll et al., 2012). According to the former hypothesis, the direct pathway is required for gating information into PFC, where it is then maintained independent of direct-pathway activity. According to the latter hypothesis, in contrast, maintenance of working-memory content requires reverberation of activity in cortico-BG-thalamic loops, explicitly involving the direct pathway. Empirical evidence does not clearly favor one interpretation over the other. The former hypothesis predicts a phasic change in direct-pathway activity only while working-memory content is gated, while the latter predicts sustained activity over delay periods of working-memory tasks. In favor of the latter hypothesis, a subset of striatal neurons has been empirically shown to exhibit sustained activities over delay periods of a spatial delayed response task (Cromwell and Schultz, 2003) and of a delayed saccade task (Hikosaka et al., 1989). In favor of the former hypothesis, however, the caudate nucleus of the striatum has been found more active during working memory manipulation than during working memory maintenance in a functional magnetic resonance imaging (fMRI) study (Lewis et al., 2004).

### 4.6. Dimensionality reduction

The direct pathway has been proposed to perform the dimensionality reduction process outlined in section 3.5. No other BG pathway is assumed to take part in this process.

## 5. Proposed functions of long and short indirect pathways

Two indirect pathways have been described, both of which inhibit cortical activity (Figure 1): a short route passes from GPe directly to GPi and arborizes there rather sparsely, while a longer route passes from GPe to STN and further from STN to GPi where it arborizes rather profusely (section 2.3). High dopamine levels foster LTD in cortico-striatal synapses that belong to these indirect pathways, while low dopamine levels facilitate LTP (section 2.4). As most models implement only one of the two indirect pathways, their interpretations might not be specific to the particular pathway included. To highlight the often-neglected fact that these pathways might establish entirely different functions, however, we nevertheless explicitly differentiate between the two pathways.

### 5.1. Global inhibition of motor programs (long indirect pathway)

In early non-computational models, the long indirect pathway is assumed to globally inhibit motor behavior (Albin et al., 1989; DeLong, 1990). This hypothesis is in good agreement with the long indirect pathway's relatively global effects on GPi as outlined in section 2.3. Functional evidence for this hypothesis comes from an optogenetic study, where stimulation of striatal MSNs of the indirect pathways resulted in decreased motor initiation and increased bradykinesia (Kravitz et al., 2010). However, this study did not differentiate between long and short indirect pathways. Moreover, striatal MSNs of the indirect pathways were stimulated relatively globally which may be expected to cause global effects on behavior even if the long indirect pathway does not act as globally as hypothesized.

### 5.2. Inhibition of specific motor programs (short indirect pathway)

Based on its sparse arborization, the short indirect pathway has been suggested to inhibit specific motor programs (Brown et al., 2004; Frank et al., 2004; Frank, 2005; Schroll et al., 2013). More specifically, it has been hypothesized to *learn* this inhibition based on unfavorable outcomes, including omissions of expected rewards (e.g., Brown et al., 2004; Schroll et al., 2013) and aversive events (Frank et al., 2004). The chain of events between the occurrence of these unfavorable outcomes and the inhibition of motor programs is assumed to be the following: unfavorable outcomes cause phasic reductions in BG dopamine levels, which then activate the short indirect pathway to suppress the response that had resulted in the unfavorable outcome. Empirically, phasic decreases in dopamine have indeed been shown to strengthen cortico-striatal synapses of the indirect pathways (Shen et al., 2008). Moreover, it has indeed been shown that omissions of expected rewards result in phasic decreases in dopamine activity (Hollerman and Schultz, 1998); it thus appears plausible that the short indirect pathway inhibits responses based on omissions of expected rewards. The consequences of aversive events, however, might be more complex: while some SNc neurons indeed become less active following aversive events, others increase their activity (Matsumoto and Hikosaka, 2009). Insofar as SNc neurons respond differently to reward omissions and aversive events, it remains speculative if the short indirect pathway inhibits responses based on aversive events as well. In favor of such an effect, blocking of the indirect pathways (but not the direct pathway) in genetically modified mice has been shown to result in impaired shock avoidance (Hikida et al., 2010). Moreover, Frank et al. (2004) showed that Parkinson's disease patients (who suffer from dopamine loss) learn better from negative as opposed to positive outcomes than healthy controls (but see Shiner et al., 2012, for a challenge of their conclusions). Thus, the short indirect pathway may well learn to inhibit motor programs based both on omissions of rewards and on aversive events.

Omissions of expected rewards occur primarily during reversal learning and extinction (i.e., when expected stimulus-response-reward associations are no longer valid). In the model by Schroll et al. (2013), therefore, the short indirect pathway inhibits specific responses specifically during reversal learning. Pharmacological studies indeed show that D2 receptor agonists (which predominantly target indirect pathways; cf. section 2.4) impair reversal learning in humans (Mehta et al., 2001). Also, D2-type receptor *ant*agonists (but not D1-type antagonists) result in reversal-learning deficits in non-human primates (Lee et al., 2007). By assuming that both D2 agonists and D2 antagonists render D2 receptors insensitive to phasic changes in physiologically generated dopamine signals, these findings are in line with Schroll et al.'s (2013) hypothesis: according to their model, suppression of previously correct responses during reversal learning requires task-related, phasic unbinding of dopamine at D2 receptors after omissions of expected rewards. Both D2 agonists and D2 antagonists can be assumed to impair such a task-related unbinding. None of the above-cited studies, however, distinguished between long and short routes of the indirect pathway. It thus remains to be established to what extent it is indeed the short route that inhibits specific responses during reversal learning.

### 5.3. Termination of executed responses (long indirect pathway)

In consideration of the long indirect pathway's relatively slow conduction velocity (section 2.2), this pathway has been hypothesized to provide termination signals for motor execution (Nambu, 2004). In line with this hypothesis, GPi cells show a triphasic change of activity in response to cortical stimulation: an early excitation via the hyperdirect pathway is followed by a brief inhibition via the direct pathway and by a late excitation via the long indirect pathway (Figure 2B). According to the termination hypothesis, the late excitation terminates those motor responses that are initiated via the intermediate inhibition. Acknowledging the long indirect pathway's relatively broad effects on GPi (section 2.3), the proposed termination signal has been assumed to act relatively globally (Nambu, 2004).

The global-response-termination hypothesis is well compatible with the hypothesis that the long indirect pathway globally inhibits responses (section 5.1). Response termination, however, requires a delay in suppression such that responses can be initiated first.

We do not know of any direct functional evidence for a termination function of the long indirect pathway.

### 5.4. Deferral of selected plans (short indirect pathway)

According to Brown et al. (2004), the short indirect pathway defers execution of specific selected responses until appropriate. This hypothesis is an extension of the specific-inhibition hypothesis outlined in section 5.2 with regard to the dimension of time. Response deferral is assumed to be no built-in function of the short indirect pathway, but needs to be learned from omissions of expected rewards. In other words, the default is to not defer chosen plans. If, however, premature release of a response via the direct pathway results in reward omission, the short indirect pathway learns to inhibit this response (Brown et al., 2004). According to the model, response deferral is learned in a context-based way by associating the deferral to any stimulus input that might be present during the deferral period. Thalamo-striatal feedback to the short indirect pathway is hypothesized to inform the short indirect pathway which response was selected before reward omission so that exactly this response can be inhibited. We do not know of any empirical evidence corroborating the deferral hypothesis.

### 5.5. Surround-inhibition of competing motor programs

Both indirect pathways have been hypothesized to establish a surround inhibition of unwanted motor programs during motor responding (Mink, 1996; Stocco et al., 2010). Mink (1996) proposed a particular role of the STN in this respect, thus referring to the long indirect pathway (but also to what is known today as the hyperdirect pathway). In contrast, Stocco et al. (2010) proposed that the short route of the indirect pathway is involved. Neither Mink (1996) nor Stocco et al. (2010) hypothesized on how broad the “space” of suppressed competing motor programs may be (i.e., if every other motor program would be inhibited or just a set of particularly strong competitors). Therefore, arborization patterns do not provide any consistent clue whether an involvement of the short or the long route is more realistic. As a challenge to both hypotheses, however, the effects of dopamine on long-term plasticity in cortico-striatal MSNs of the indirect pathways are exactly opposite those observed in cortico-striatal MSNs of the direct pathway (section 2.4; Shen et al., 2008). Thus, when facilitation of a response via the direct pathway is strengthened by dopamine, surround inhibition of its competitors may not be strengthened as well; center facilitation and surround inhibition can not be established at the same time unless the activities of dopamine neurons that target the “center” increase, while those that target the “surrounds” may simultaneously decrease. Since such an effect has not yet been reported, the hyperdirect pathway might be a more suitable candidate for surround inhibition than any of the indirect pathways (cf. section 6.4).

### 5.6. Control system

Challenging the subdivision of BG fiber tracts into direct, indirect and hyperdirect pathways, BG have been proposed to consist of a selection pathway, containing what is referred to as direct and hyperdirect pathways in this review, and of a control pathway, vaguely consisting of what is termed long and short indirect pathways here (Gurney et al., 2001a,b; Humphries et al., 2006). More specifically, the control pathway is assumed to consist of the full short indirect pathway, the long indirect pathway up to the STN and an additional route from cortex via STN to GPe (Gurney et al., 2001a). According to Gurney et al. (2001a,b), the control pathway does not have a separable function itself, but rather supports direct and hyperdirect pathways in selecting responses. Among its functions, the control pathway is hypothesized to regulate the amount of activity in STN (and thereby also in GPi): according to the model, motor selection requires that the amount of global motor inhibition is neither so strong that it overrules any facilitation of a specific motor program via striatum, nor so weak that multiple responses are selected simultaneously. By regulating the activity of STN cells and thereby the amount of global motor inhibition, the control pathway ensures an appropriate balance between excitation and inhibition such that neither too many nor too few responses are released simultaneously.

A similar concept is based on the architecture of direct and long indirect pathways as specified in this review (cf. Figure 1): in Suri et al.'s (2001) model, the long indirect pathway is hypothesized to globally increase motor inhibition such that only significant contributions of the direct pathway will result in cortical activation, while insignificant direct-pathway effects will be suppressed.

### 5.7. Closing the gate to working memory (short indirect pathway)

With regard to working memory, the short indirect pathway has been proposed to prevent gating of information into working-memory storage systems (O'Reilly and Frank, 2006). According to this hypothesis, the short indirect pathway and the direct pathway oppose each other in a push-and-pull manner (section 7.1) to forbid or allow gating of information into working memory, respectively. The hypothesis is structurally similar to, and therefore compatible with, the idea that the short indirect pathway inhibits (gating of) specific motor programs (section 5.2); these functions could be performed by different cortico-BG-thalamic loops. Although there is evidence for BG involvement in working-memory functions (Levy et al., 1997; Lewis et al., 2004; Landau et al., 2009), there is, to the best of our knowledge, no data on the specific gating function of the short indirect pathway proposed by O'Reilly and Frank (2006).

## 6. Proposed functions of the hyperdirect pathway

As outlined in sections 2.2 and 2.3, the hyperdirect pathway excites GPi relatively fast and relatively globally. A major proportion of its inputs derives from axon collaterals of pyramidal tract neurons (section 2.1), while synaptic plasticity in this pathway is not yet well understood (section 2.4).

### 6.1. Prevention of premature responses

Based on its fast and relatively global effects on GPi, the hyperdirect pathway has been proposed to globally prevent premature responses until response selection has been completed (Frank, 2006; Stocco et al., 2010). Along these lines, the hyperdirect pathway has been predicted to be particularly vital in situations where extensive response conflict occurs (Frank, 2006; Frank et al., 2007), i.e., whenever multiple competing motor programs are simultaneously active in premotor cortex. Recordings of STN activity during high-conflict and low-conflict choices have been performed to investigate this prediction. A typical paradigm starts with a couple of training trials, in which subjects are presented with pairs of stimuli (e.g., A-B or C-D) and have to choose one stimulus of each pair. Each stimulus is associated with a fixed reward probability across trials (e.g., A: 20%—B: 80%, and C: 30%—D: 70%).

Being instructed to maximize rewards, subjects are supposed to learn to choose the stimulus of each pair that provides better average outcomes (i.e., B and D respectively, in our example). In subsequent test trials, pairs are shuffled such that high-conflict pairs (e.g., B–D) and low conflict pairs (e.g., A–D) may be presented. Using such a task, human patients ON deep brain stimulation (DBS) of the STN (which inhibits spiking activity in STN, Gradinaru et al., 2009, and thus eliminates task-related information processing in this nucleus) have been shown to make faster decisions under high response conflict than patients OFF DBS (Frank et al., 2007). Moreover, intracranial EEG recordings from DBS electrodes have revealed differences in STN oscillatory activity between high-conflict and low-conflict trials (Cavanagh et al., 2011), thus arguing for a contribution of STN to conflict processing, in line with Frank et al.'s (2007) prediction.

### 6.2. Stopping prepared responses before execution

Along similar lines, the hyperdirect pathway has been hypothesized to globally inhibit prepared responses when a stop signal is shown before response execution (Aron and Poldrack, 2006; Aron, 2011; Wiecki and Frank, 2013). This hypothesis is fully compatible with the premature-response-inhibition hypothesis outlined in section 6.1; the hyperdirect pathway may flexibly switch between both of these functions as required by context. Both functions require fast and global inhibition of motor programs, which would fit well with the hyperdirect pathway's fast conduction velocity and its relatively global effects on GPi. Indeed, not just the premature-response-inhibition hypothesis, but also the stop hypothesis is corroborated by empirical evidence: in an fMRI study, STN (which is part of the hyperdirect pathway—but also of the long indirect pathway) has been shown to be more active in humans in stop trials than in go trials of a stop-signal task (Aron and Poldrack, 2006). In that same study, STN has been found more active in subjects that show a fast inhibition of responses after a stop cue (i.e., a fast stop-signal reaction time, SSRT) than in subjects with slow response inhibition. Contrarily, however, another fMRI study reported smaller STN activity in fast-inhibiting subjects than in slow inhibitors (Ray Li et al., 2008). As a challenge to the stop-signal hypothesis, PD patients (whose STN activity is increased; Kreiss et al., 1997; Huang et al., 2007), show slower (instead of faster) inhibition of responses in stop-signal tasks (Gauggel et al., 2004).

### 6.3. Deactivation of BG to allow for top-down control

The hyperdirect pathway has been hypothesized to subdue BG influences on motor cortex in order to allow for top-down PFC control over motor-cortical activities (Chersi et al., 2013). According to this hypothesis, PFC inputs to the hyperdirect pathway decrease activities of all GPi/ SNr neurons to similar levels via inhibitory interneurons, thereby overruling any response-activating effects caused by the direct pathway and preventing task-related BG outputs (Chersi et al., 2013). PFC may then control motor-cortical activities itself. By proposing that the hyperdirect pathway globally overrules any effects of the direct pathway, the deactivation hypothesis has a common assumption with the premature-response-inhibition hypothesis (section 6.1) and the response-stopping hypothesis (section 6.2). However, the deactivation hypothesis specifies that the hyperdirect pathway *decreases* activities in BG output nuclei (via inhibitory interneurons; Chersi et al., 2013), while the other two hypotheses assume it to increase firing in these nuclei. Since the major effect of the hyperdirect pathway on GPi/SNr is known to be excitatory (cf. Figure 1), we do not consider the deactivation hypothesis to be particularly plausible in this respect. It might, however, still hold true in its core: a global *increase* in GPi firing could deactivate BG to allow for top-down control just as well.

### 6.4. Surround inhibition of competing motor programs

Just like the indirect pathways (section 5.5), the hyperdirect pathway has been hypothesized to establish surround-inhibition of unwanted motor programs during responding (Gurney et al., 2001a; Nambu, 2004; Humphries et al., 2006; Schroll et al., 2013). Two versions of this hypothesis exist: according to the first, the hyperdirect pathway inhibits only those responses that compete for execution with the desired response, but not the desired response itself which is instead facilitated via the direct pathway (Schroll et al., 2013). According to the second version, the hyperdirect pathway globally inhibits all motor programs, including the desired one, which is distinguished only by its additional activation via the direct pathway (Gurney et al., 2001a; Nambu, 2004; Humphries et al., 2006).

Both hypotheses well reflect the different arborization patterns of direct and hyperdirect pathways, which have been found sparse and profuse, respectively (section 2.3). We don't know of any empirical study investigating the strict surround-inhibition hypothesis against the more unspecific surround-inhibition hypothesis.

### 6.5. Working-memory update

The hyperdirect pathway has been hypothesized to clear information from working-memory and to thus allow for updating of working-memory content (Schroll et al., 2012). According to this hypothesis, the hyperdirect pathway breaks reverberation of activity in cortico-BG-thalamic loops (which is assumed to be the neuronal basis of working-memory maintenance, section 4.5). Empirical evidence for this hypothesis is yet rather unspecific: DBS of the STN in Parkinson's disease patients (which is assumed to inhibit spiking activity in this nucleus; Gradinaru et al., 2009) impaired working-memory performance in a spatial delayed response task (Hershey et al., 2008) and in an n-back task (Alberts et al., 2008); to what extent these effects were in fact produced by failures in updating working-memory content, however, or may have been caused by other types of errors, was not delineated. The working-memory-update hypothesis may be generalized to an involvement of the hyperdirect pathway in updating of any information that may be maintained in closed cortico-BG-thalamic loops.

## 7. Proposed interaction patterns between pathways

Having reviewed prominent hypotheses on pathway functions, we will now outline in how far these hypotheses may be grouped into general “principles” of pathway functions. In section 2.5, we reviewed evidence that BG are compartmentalized into a variety of largely independent loops related to motor, premotor, cognitive, motivational, and emotional functions. Since each of these loops is assumed to contain its own separate channel of each BG pathway, we pinpointed that each pathway might contribute to a variety of different functions at the same time. We therefore concluded that it might be a more fruitful approach to search for general principles of pathway functions than for individual pathway contributions related to different loops. While such general principles may be defined from various viewpoints, we hold the view that the models reviewed in sections 3 to 6 differ most consistently from each other with regard to their assumptions on how pathways interact in their effects on cortex. While most models agree that the direct pathway somehow activates specific cortical representations, assumptions on how indirect and hyperdirect pathways interact with this activation are more controversial. In the following sub-sections, we will outline these different concepts.

Importantly, different concepts of pathway interactions are not always mutually exclusive. Rather, the BG might learn from reinforcements which patterns of interactions are most appropriate under different circumstances and might thereby flexibly adapt information processing to environmental demands.

### 7.1. Push-and-pull opposition

**Figure 4:**
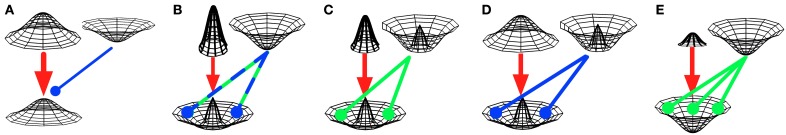
**Hypotheses on interactions between pathway outputs.** Three-dimensional Gaussians depict neuronal activities (z-axis), as elicited by basal-ganglia pathways, for “central” and “surrounding” cortical representations (x- and y-axes). Direct-pathway effects are denoted by red arrows, while the effects of hyperdirect and indirect pathways are denoted by green and blue arrows, respectively. Pointed arrows denote excitatory, rounded arrows inhibitory effects. **(A)** Push-and-pull opposition: direct and short indirect pathways may oppose each other in a push-and-pull manner, where the effects of direct and short indirect pathways are equal in spatial extent (e.g., Brown et al., 2004; Frank, 2005; Schroll et al., 2013). In the example shown here, the direct pathway (thick red arrow) overpowers the short indirect pathway (thin blue arrow). **(B)** Center-surround cooperation. The direct pathway activates specific cortical representations, while either the hyperdirect pathway (Nambu, 2004) or the long indirect pathway (Mink, 1996) globally inhibit these representations. Since the direct pathway's effect is assumed to be more powerful, center-surround activation emerges. **(C)** Strict center-surround cooperation. The direct pathway activates specific cortical representations, while the hyperdirect pathway inhibits surrounding (i.e., “competitive”) representations, but not the activated representation itself (Schroll et al., 2013). The resulting effect is mostly equivalent to **(B)**. **(D)** Center-surround cooperation with global activation. The direct pathway excites cortex relatively globally, while the short indirect pathway inhibits all but the “center” representation (Stocco et al., 2010). As a result, again, the central cortical representation is activated, while its “surrounds” are inhibited. **(E)** Global blocking of activation. The direct pathway tries to activate specific cortical representations, while the hyperdirect pathway globally inhibits them. In contrast to **(B)**, the hyperdirect pathway is more powerful than the direct pathway and thus overrules any direct-pathway effect (cf. Aron and Poldrack, 2006; Frank, 2006). Please note that pathway effects are depicted as Gaussians for merely illustrative purposes. Most models do not implement Gaussian functions, but rather assume “box-car” (i.e., all-or-nothing) effects.

Direct and short indirect pathways have been hypothesized to oppose each other in a push-and-pull manner (Figure 4A; e.g., Brown et al., 2004; Frank, 2005; O'Reilly and Frank, 2006): while the direct pathway is assumed to activate specific cortical representations, the short indirect pathway might at the same time try to inhibit them. The relative balance between activation and inhibition might then determine if a particular representation is activated or inhibited overall. The direct pathway's activation is usually assumed to be strengthened by dopamine bursts (i.e., by unexpectedly strong reinforcements; Hollerman and Schultz, 1998), while the short indirect pathway's inhibition is assumed to be strengthened by dopamine dips that might either derive from omissions of expected rewards (Brown et al., 2004; Schroll et al., 2013) or from punishments (e.g., Frank et al., 2004).

The push-and-pull assumption underlies the specific-response-inhibition hypothesis outlined in section 5.2, the response-deferral hypothesis outlined in section 5.4 and the gate-closing hypothesis outlined in section 5.7.

### 7.2. Center-surround cooperation

Direct and hyperdirect pathways have been proposed to establish a center-surround system of activation and inhibition, where the direct pathway activates specific “central” cortical representations, while the hyperdirect pathway inhibits “surrounding” (i.e., competitive) representations (Nambu, 2004; Schroll et al., 2013). As outlined in section 6.4, different models assume the hyperdirect pathway to either inhibit only competing motor programs, sparing the center (Figure 4C; Schroll et al., 2013), or to inhibit the center-facilitated motor program as well (Figure 4B; Nambu, 2004). According to the latter hypothesis, the direct pathway needs to be powerful enough to overrule any effect by the hyperdirect pathway to still activate the “center” representation. According to the former hypothesis, center-activation and surround-inhibition may partially compensate for each other: by activating the center more strongly, surround inhibition may become less relevant, while strong surround inhibition may require less powerful center-activation.

Center-surround collaboration has been proposed for direct and indirect pathways as well (section 5.5). Mink (1996) assumed direct and long indirect pathways to interact as depicted in Figure 4B, while Stocco et al. (2010) proposed a different mechanism: according to their model, the direct pathway activates cortex relatively globally (i.e., unspecifically), while the short indirect pathway inhibits all undesired representations, resulting in activation of only the desired representation (Figure 4D).

### 7.3. Global blocking of activations

According to a different set of hypotheses, global inhibition of cortical representations via the hyperdirect pathway is powerful enough to overrule any specific activation caused by the direct pathway (Figure 4E). Whenever the hyperdirect pathway globally inhibits cortical representations, thus, the direct pathway becomes powerless. Such a function of the hyperdirect pathway underlies the premature-response prevention hypothesis reviewed in section 6.1, the response-stopping hypothesis outlined in section 6.2 and the working-memory-update hypothesis outlined in section 6.5. By approximation, it also underlies the deactivation hypothesis reviewed in section 6.3, which, however, states that the hyperdirect pathway overrules any direct-pathway effects by globally *facilitating* activation of cortical representations.

### 7.4. Modulation of activation

An again different set of hypotheses (section 5.6) suggests that the long indirect pathway modulates the direct pathway's effects. Suri et al. (2001) proposed that the long indirect pathway globally inhibits cortical representations to such an extent that only strong activations of specific desired representations via the direct pathway result in cortical activity, while weak activations will be suppressed (Suri et al., 2001). According to this hypothesis, thus, the direct pathway may overrule the long indirect pathway's effects only if it is powerful. The hypothesis thus lies somewhere between the center-surround cooperation as depicted in Figure 4B and the global-blocking hypothesis as shown in Figure 4E. In a functionally related proposal (section 5.6), a control pathway (vaguely consisting of the two indirect pathways; Gurney et al., 2001a,b; Humphries et al., 2006) controls the number of cortical representations that can be activated simultaneously by a similar process, as outlined in section 5.6.

## 8. Future directions: tests of model assumptions

Conflicting hypotheses on pathway functions may be empirically tested against each other. Critically, such tests will have to link brain processes to overt behavior and will thus have to be performed in awake animals or humans. In the following sub-sections 8.1 to 8.3, we suggest a few such experiments.

### 8.1. The direct pathway: update vs. maintenance of working-memory content

Models are relatively unanimous about the direct pathway's functional contribution to motor responding. With regard to its potential involvement in working-memory processes, however, two relatively incompatible hypotheses have been proposed (section 4.5): according to the first, the direct pathway takes part in gating working-memory content (Gruber et al., 2006; O'Reilly and Frank, 2006), while working-memory maintenance is subserved by the cortex. According to the second, the direct pathway contributes to working-memory maintenance, while working-memory updating is ensured by the hyperdirect pathway. Please note, however, that a direct-pathway involvement in both maintenance and updating of working memory content is conceivable: the direct pathway could, for instance, contribute to working-memory maintenance in closed loops and to working-memory updating in interlinked open loops.

To test the maintenance against the updating hypothesis, an experimenter could inactivate direct-pathway MSNs in genetically modified mice (cf. Hikida et al., 2010) and observe the effects of this manipulation on working-memory performance. If the direct pathway is involved in gating of information, but not in its maintenance, updating of working-memory content should be impaired, while there should be no loss of information over time once the information is correctly gated into working memory. In brief, thus, errors should mostly be of the perseverative type. If, in contrast, the direct pathway takes part in maintenance of information, gating might be relatively unimpaired, but working-memory content should decay over time. Animals should then show relatively random (rather than perseverative) errors. The experimenter might want to inactivate direct-pathway MSNs during *training* of working-memory tasks, since this might more consistently involve BG participation than an already automatized task (cf. Antzoulatos and Miller, 2011; Waldschmidt and Ashby, 2011).

Related studies could also be performed with human subjects: rather than *inactivating* the direct pathway, however, natural variances in direct-pathway gene expression could be related to working-memory performance (cf. Heinz et al., 1996, for such a study outside the context of working memory).

### 8.2. The short indirect pathway: reversal learning vs. avoidance of aversive events

It has been proposed that the short indirect pathway learns response inhibition based on aversive events (Frank et al., 2004). Alternatively, the pathway has been hypothesized to learn inhibition based on omissions of expected rewards, which occur consistently during reversal learning and extinction (Schroll et al., 2013). While these functions do not in principle contradict each other, none is yet firmly established empirically. An experimenter could design a stimulus-response learning paradigm. In a first phase of this experiment, animals would learn associations between stimuli and responses (i.e., button presses) based upon either rewards that are presented when the correct button is chosen or punishments that are presented when the incorrect button is chosen. In a second phase, previously learned stimulus-response associations would be reversed or extinguished. Measures of the short indirect pathway's strength would be recorded over the learning process (e.g., magnitudes of phasic firing-rate decreases in GPe during responding). The aversive-events hypothesis predicts that phasic decreases in GPe activity should become stronger after each aversive event, whereas the reward-omission hypothesis predicts that phasic decreases should become stronger during reversal of rewarded associations or during extinction.

### 8.3. Surround inhibition: long indirect vs. short indirect vs. hyperdirect pathway

While some authors hypothesize surround-inhibition of unwanted motor programs to be implemented via long or short indirect pathways (section 5.5; Mink, 1996; Stocco et al., 2010), others hypothesize the hyperdirect pathway to control such a function (section 6.4; Nambu, 2004; Schroll et al., 2013). While the mechanisms that are assumed to establish surround inhibition differ between models, their effects are mostly equivalent (Figures 4B–D): A “central” cortical representation is activated, while its surrounding representations (i.e., competitors) are suppressed. To find out which pathway (if any) is responsible for such a surround-inhibition, an experimenter could measure GPi firing rates in intact, GPe-lesioned and STN-lesioned animals during performance of clearly defined motor responses that have easily identifiable competitors (such as moving a limb toward left vs. right). The surround-inhibition hypothesis of unwanted motor programs implies that those GPi neurons, which show a phasic decrease in activity with response *A*, show an increase in activity during competitive response *B* and that there are other neurons that behave vice versa. If the long indirect pathway is responsible for such a surround inhibition, lesions of STN and GPe should each eliminate the phasic increase in GPI activity. If, however, the short indirect pathway is involved, only lesions of GPe should eliminate it. If the hyperdirect pathway is critical, only STN lesions should abolish the phasic increase in firing.

## 9. Future directions: model developments

Although existing models of BG pathways account for a variety of anatomical, physiological and biochemical data, some major findings have not yet been implemented at all. Table 2 contains some of these findings and specifies how computational modeling might help to understand their significance with regard to the functions of BG pathways.

**Table 2:** **Perspectives of computational modeling**.

**Significant empirical findings that have not yet been implemented in computational models**	**What computational modeling might contribute to the understanding of these findings**
Aversive events cause increased activity for some, but decreased activity for other dopamine neurons (Matsumoto and Hikosaka, 2009).	How do aversive events affect membrane potentials, firing rates and synaptic plasticity in BG pathways?
Striatal MSNs of the direct pathway develop en-passant synapses in the GPe, which is part of the indirect pathway (Lévesque and Parent, 2005).	What is the functional role of these en-passant synapses? What may be their rules of synaptic plasticity?
Input signals to the BG derive not only from cortex, but also from thalamus (Berendse and Groenewegen, 1990; Lanciego et al., 2004).	How do cortical and thalamic input signals to BG differ?
STN contains both D1 and D2 dopamine receptors in considerable quantities (Flores et al., 1999).	How do D1 and D2 receptors in STN contribute to synaptic plasticity in hyperdirect and long indirect pathways?
Striatal MSNs receive inputs from several types of striatal interneurons (Kawaguchi, 1993; Tepper, 2010).	How do these various interneurons contribute to the functions of direct and indirect BG pathways?

Some empirical findings have not yet been implemented in computational models. We here highlight some of these findings and outline what computational modeling might contribute to their understanding.

It may be noted that Table 2 repeatedly relates to synaptic plasticity. We hold the view that the mechanisms of synaptic plasticity in BG pathways are a key to understanding their functions. Many computational models rely on the assumption that BG vitally contribute to reinforcement learning. If this is correct, the pathways' mechanisms of synaptic learning must be of central importance. To date, however, only cortico-striatal plasticity has been investigated extensively (e.g., Shen et al., 2008), whereas potential mechanisms of plasticity in cortico-subthalamic, striato-pallidal, subthalamo-pallidal, and striato-striatal synapses remain elusive. Because of these knowledge gaps, computational models differ extensively in their assumptions on the mechanisms of synaptic plasticity in BG pathways. Combined empirical and modeling efforts will be required to unveil these mechanisms and to analyze how they contribute to the functions of BG pathways. Neuro-computational modeling in particular might be used to investigate how synaptic plasticity controls the emergence of pathway functions. Schroll et al. (2013) recently showed that by specifying the rules of synaptic plasticity in a computational model of BG pathways (but not the pathways' patterns of connectivity), pathway functions self-organized as the model learned a behavioral task from reinforcements. Such an approach of specifying plasticity and investigating the emergence of pathway functions could be repeated for refined mechanisms of plasticity (accounting for instance, for spike-time-dependent effects) and extended to BG fiber tracts that are no core elements of BG pathways (like striatal interneurons or the back-projections from GPe to the striatum). Different model may be compared against each other by analyzing their performance on relevant behavioral paradigms.

## 10. Conclusions

In the Introduction we posed the question, why BG contain such a multitude of nuclei and fiber tracts. By reviewing influential hypotheses on the functions of BG pathways, we hope to have outlined potential functional advantages of such complexity. It has to be admitted, however, that all of the interpretations reviewed have been developed from a reverse-engineering standpoint, asking why BG are complex, given that this is the case. They do not answer why complexities evolved in the first place or if there might have been simpler solutions that would have guaranteed equivalent functionality.

Most theorists assume that BG nuclei and fiber tracts give rise to separate pathways and that these pathways fulfill distinct functions. While they mostly agree that the direct BG pathway activates specific cortical representations, the functions of indirect and hyperdirect pathways are under intense debate. We have outlined various hypotheses on these pathways' functions and suggested that they may be grouped according to these pathways' hypothesized interactions with the direct pathway. Specifically, we have identified push-and-pull opposition, center-surround cooperation, global blocking of direct-pathway effects and modulation of direct-pathway effects as major proposed interaction patterns.

We hope to have motivated stringent empirical tests of hypotheses on pathway functions, believing that theory-based research promises exciting advances in the understanding of BG complexity.

### Conflict of interest statement

The authors declare that the research was conducted in the absence of any commercial or financial relationships that could be construed as a potential conflict of interest.
